# Continuous control of tracheal cuff pressure: an effective measure to prevent ventilator-associated pneumonia?

**DOI:** 10.1186/s13054-014-0512-2

**Published:** 2014-09-06

**Authors:** Anahita Rouzé, Emmanuelle Jaillette, Saad Nseir

**Affiliations:** Pôle de Réanimation, Hôpital Roger Salengro, CHRU, rue E. Laine, 59037 Lille, cedex France; EA 2694, Université Nord de France, 1 Place de Verdum, 59000 Lille, France

**Keywords:** ᅟ

## Abstract

In a previous issue of *Critical Care*, Lorente and colleagues reported the results of a prospective observational study aiming at evaluating the effect of continuous control of cuff pressure (P_cuff_ ) on the incidence of ventilator-associated pneumonia (VAP). The results suggest a beneficial impact of this intervention on VAP prevention, which is in line with the results of a recent randomized controlled study. However, another randomized controlled study found no significant impact of continuous control of P_cuff_ on VAP incidence. Several differences regarding the device used to control P_cuff_, study population, and design might explain the different reported results. Future randomized multicenter studies are needed to confirm the beneficial effect of continuous control of P_cuff_ on VAP incidence. Furthermore, the efficiency and cost-effectiveness of different available devices should be compared. Meanwhile, given the single-center design and the limitations of the available studies, no strong recommendation can be made regarding continuous control of P_cuff_ as a preventive measure of VAP.

## Introduction

In a previous issue of *Critical Care*, Lorente and colleagues [1] reported the results of a prospective study aiming at evaluating the impact of continuous control of cuff pressure (P_cuff_) on the incidence of ventilator-associated pneumonia (VAP). They included a large number of patients intubated and ventilated for more than 48 hours (150 patients in a continuous control group and 134 patients in a routine care group). The target P_cuff_ was 25 cm H_2_O in both groups. Whereas the main clinical characteristics were similar in the study groups, VAP rate was significantly reduced by the use of continuous control of P_cuff_. Additionally, continuous control of P_cuff_ and use of subglottic secretion drainage were independently associated with reduced incidence of VAP. Kaplan-Meier analysis showed a significantly higher proportion of patients remaining free from VAP using continuous control of P_cuff_ compared with intermittent control using a manometer.

**Table 1:** **Characteristics of studies assessing the impact of continuous control of cuff pressure on ventilator-associated pneumonia incidence**

	Valencia *et al*. [5] (2007)	Nseir *et al*. [4] (2011)	Lorente *et al*. [1] (2014)
Number of included patients	142	122	284
Type of study	Randomized controlled	Randomized controlled	Prospective cohort
Primary objective	VAP	Microaspiration	VAP
Device	Electronic	Pneumatic	Electronic
Target P_cuff_, cm H_2_O	25	25	25
Surgical patients, %	28	0	28
Chronic respiratory disorders, %	38	27	15
Oral care	CHX 0.12% X3/d	CHX 0.10% X3/d	CHX 0.12% X3/d
Semirecumbent position	Yes	Yes	Yes
VAP incidence in control group, %	15	26	22
Reduction in VAP rate, %	NS	62	51
% P_cuff_ 20-30 cm H_2_O in intervention group	79	98	100

CHX, chlorhexidine; NS, not significant; P_cuff_, cuff pressure; VAP, ventilator-associated pneumonia; X3/d, 3 times a day.

## Comparison with previous studies

The strengths of this study are the large number of included patients and adjustment for confounders using Cox proportional analysis. As acknowledged by the authors, the absence of randomization and blinding is one of the limitations of the study. In addition, the impact of continuous control of P_cuff_ on tracheal ischemic lesions was not evaluated. However, previous animal and clinical studies found no significant effect of this intervention on the incidence of intubation-related tracheal damage [2,3]. Furthermore, efficiency of the electronic device used by the authors in continuously controlling P_cuff_ was not previously evaluated in critically ill patients.

The lower rate of VAP in patients who received continuous control of P_cuff_ is in line with the results of a recent randomized controlled study performed by our group [4], although the primary outcome of that study was not VAP but abundant microaspiration of gastric content. Another randomized controlled study found no significant impact of continuously controlling P_cuff_ on VAP incidence [5]. Several differences regarding the device used to control P_cuff_, study population, and design might explain the different reported results (Table 1).

## Microaspiration and underinflation of tracheal cuff

Microaspiration of contaminated oropharyngeal and gastric secretions is the main mechanism of entry of bacteria into the lower respiratory tract [6]. Recently, several markers of microaspiration, including pepsin and salivary amylase, were described and validated [7–9]. Interestingly, these markers are quantitative, allowing accurate evaluation of microaspiration. It is well known that subsequent development of VAP is tightly correlated to the quantity of bacteria present in the lower respiratory tract [10]. The use of these biomarkers in critically ill patients could be helpful in evaluating the efficiency of a new device aiming at reducing the incidence of VAP via the reduction of microaspiration, before conducting large multicenter studies to test the effect of such a device on VAP incidence.

Underinflation of the tracheal cuff is usually defined as P_cuff_ of less than 20 cm H_2_O and was identified by one prospective observational study as an independent risk factor for VAP in a subgroup of patients without antimicrobials [11]. However, microaspiration could occur at higher P_cuff_ depending on tracheal anatomy and patient movements [12]. In fact, microaspiration is a multifactorial process related to mechanical ventilation, tracheal tube, enteral nutrition, and general factors. Therefore, to prevent microaspiration and subsequent VAP, all of these factors, not just P_cuff_, should be taken into account.

## Current practice and future studies

In spite of routine manual control of P_cuff_ using a manometer, intubated critically ill patients spend a large amount of time with underinflation and overinflation (>30 cm H_2_O) of the tracheal cuff. In a cohort of 101 critically ill patients intubated with a polyvinylchloride-cuffed tube, P_cuff_ was continuously recorded for 8 hours after manual adjustment of P_cuff_ at 25 cm H_2_O [13]. Only 18 % of study patients spent 100 % of recording time with a normal (20 to 30 cm H_2_O) P_cuff_. Fifty-four percent of study patients developed cuff underinflation, 73 % developed cuff overinflation, and 44 % developed both. Subsequent studies reported similar results in patients intubated with polyurethane-cuffed tracheal tubes [14,15]. Several devices aiming at continuously controlling P_cuff_, including the one used by Lorente and colleagues, are currently available on the market. Unfortunately, the efficiency of some of these devices was never tested in clinical studies. Therefore, before these devices can be used in critically ill patients, well designed and performed studies are required.

## Conclusions

Future randomized multicenter studies should confirm the beneficial effect of continuous control of P_cuff_ on VAP incidence. Furthermore, the efficiency and cost-effectiveness of different available devices should be compared. Meanwhile, given the single-center design and the limitations of the available studies, no strong recommendation can be made regarding continuous control of P_cuff_ as a preventive measure of VAP.

## Abbreviations

P_cuff_: Cuff pressure
VAP: Ventilator-associated pneumonia
